# Trimethylamine N-Oxide Levels in Non-Alcoholic Fatty Liver Disease: A Systematic Review and Meta-Analysis

**DOI:** 10.3390/metabo12121243

**Published:** 2022-12-09

**Authors:** Panagiotis Theofilis, Aikaterini Vordoni, Rigas G. Kalaitzidis

**Affiliations:** Center for Nephrology “G. Papadakis”, General Hospital of Nikaia—Piraeus “Agios Panteleimon”, 18454 Piraeus, Greece

**Keywords:** non-alcoholic fatty liver disease, trimethylamine N-oxide, gut microbiota

## Abstract

Non-alcoholic fatty liver disease (NAFLD) represents an entity with an increasing prevalence which is characterized by significant hepatic and extrahepatic complications. Its pathophysiology is multifactorial, with gut dysbiosis being considered a major determinant. In this systematic review and meta-analysis, we tried to evaluate the association between the major gut microbial metabolite trimethylamine N-oxide (TMAO) and NAFLD. We performed a literature search for studies that determined circulating TMAO in patients with and without NAFLD. The database search identified 136 studies, and upon application of the exclusion criteria, 7 studies with 7583 individuals (NAFLD 2923, control 4660) were ultimately included in the meta-analysis. Compared to the control group, NAFLD patients had significantly higher circulating TMAO (SMD: 0.66, 95% CI −0.12 to 1.21, *p* = 0.02, I^2^: 94%). The results remained unaffected after the exclusion of one influential study. The subgroup analysis revealed significantly higher TMAO in individuals with histologically proven NAFLD and in studies measuring TMAO with high-performance liquid chromatography. No differences were observed according to the study design or study region. However, funnel plot asymmetry was observed, indicating publication bias. In conclusion, patients with NAFLD had increased levels of TMAO, a hazardous gut microbial metabolite, suggesting its important role in the gut–liver interaction.

## 1. Introduction

Non-alcoholic fatty liver disease (NAFLD) and the newly defined metabolic dysfunction-associated fatty liver disease (MAFLD) are increasingly common entities according to recent epidemiological reports, with an estimated prevalence of around 32.4% [1,2]. It should be noted that there was a trend toward greater NAFLD prevalence rates during the last decade. This alarming evidence could be attributed to the increasing frequency of its most prominent risk factors, namely diabetes mellitus and obesity [3,4]. As NAFLD occurs more frequently, its hepatic and extra-hepatic complications are expected to rise. The progression of NAFLD promotes the development of steatohepatitis and fibrosis, which could ultimately result in liver cirrhosis and hepatocellular carcinoma [5,6,7,8,9]. Moreover, individuals with NAFLD are at increased risk of cardiovascular complications, namely acute myocardial infarction [10], acute ischemic stroke [11], heart failure with preserved ejection fraction [12], and renal disease development [13].

Several pathophysiologic mechanisms have been implicated in the evolution of NAFLD. Inflammation, oxidative stress, and endothelial dysfunction are among the well-established processes that are detrimental [14,15,16]. These mechanisms may also explain the association of NAFLD with cardiorenal complications. Novel mechanistic studies point to the interaction between the gut microbiome and its disruption and the development of NAFLD. Trimethylamine N-oxide (TMAO), a prominent metabolite that has been found in increased concentrations in cases of gut dysbiosis, may serve as an important biomarker in NAFLD. Therefore, this systematic review and meta-analysis aimed to assess the difference in TMAO levels between subjects with and without NAFLD.

## 2. Materials and Methods

### 2.1. Search Strategy, Inclusion, and Exclusion Criteria

The systematic review and meta-analysis were conducted in accordance with the guidelines of the 2020 Preferred Reporting Items of Systematic Reviews and Meta-Analyses (PRISMA) statement [17], as shown in Supplementary Table S1. The study was pre-registered in the PROSPERO International prospective register of systematic reviews (registration number: CRD42022371366).

We performed a literature search in the PubMed, Scopus, and Cochrane library electronic databases from inception until 22 October 2022. We aimed to detect the English language cross-sectional, case-control, cohort, and randomized studies that evaluated the levels of TMAO in individuals with and without NAFLD. We employed the following search terms: (“trimethylamine N-oxide” OR “TMAO”) AND (“nonalcoholic fatty liver disease” OR “non-alcoholic fatty liver disease” OR “NAFLD” OR “metabolic dysfunction fatty liver disease” OR “MAFLD” OR “fatty liver” OR “fatty liver disease” OR “steatosis” OR “liver steatosis” OR “hepatic steatosis” OR “steatohepatitis” OR “nonalcoholic steatohepatitis” OR “non-alcoholic steatohepatitis” OR “NASH”). We defined the difference between the blood TMAO levels of NAFLD patients and the control group as the primary outcome of interest. We excluded studies performed in preclinical models, as well as papers that did not report NAFLD groups or TMAO values.

### 2.2. Data Extraction and Quality Assessment

The full text of the eligible studies was assessed by two independent review authors (PT, AV), who then extracted the following data: the TMAO levels in the NAFLD and the control groups, the publication year, the study design and region, the definition of NAFLD, and the TMAO measurement method and units. After the independent data extraction, cross-checking was performed in a meeting. When discrepancies were found in the data extraction, the third review author (RGK), who was blinded to the initial data, was responsible for the re-evaluation of the studies in question and for making the final decision.

All numerical, continuous data were transformed to mean ± standard deviation for the final analysis, as previously described [18]. Moreover, the calculation of the overall TMAO levels in the presence of multiple NAFLD categories was performed using validated methods [18]. The Newcastle–Ottawa Scale for the assessment of the methodological quality of studies was used as a risk of bias tool [19].

### 2.3. Statistical Analysis

We performed a meta-analysis to assess the difference in blood TMAO between individuals with and without NAFLD. Effect sizes were pooled using a random effect model, and the results were expressed as uncorrected standardized mean difference (SMD) using Cohen’s d as the effect size metric with 95% confidence intervals (CI). Between-study heterogeneity was assessed using the calculation of I^2^, with values of 25%, 50%, and 75% indicating mild, moderate, and substantial heterogeneity, respectively. We additionally performed an influence analysis followed by an updated meta-analysis with the exclusion of the influential studies. The existence of publication bias was assessed using a funnel plot inspection. The Egger’s test was not performed due to the small number of studies (*n* = 7). Furthermore, we carried out a subgroup analysis according to the NAFLD definition, the method of TMAO measurement, and the presence of a significant difference in the age and sex distributions across the examined groups. All meta-analyses were generated using the meta and dmetar packages in R studio (version 1.4.1106, Posit inc., Boston, MA, USA).

## 3. Results

### 3.1. Study Selection

**Figure 1:**
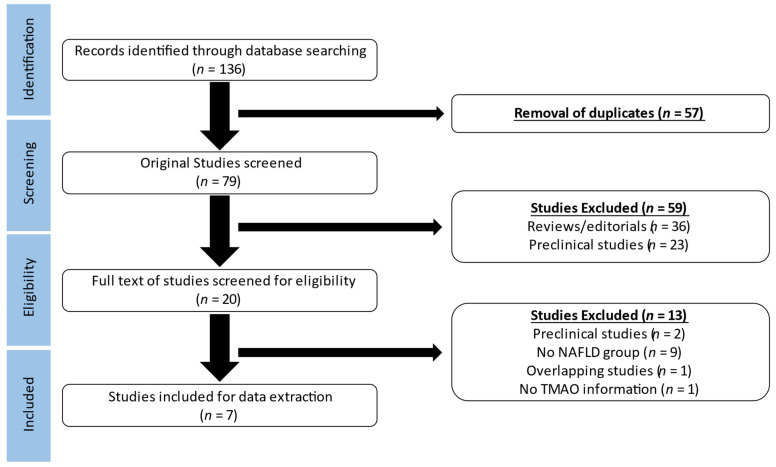
Preferred Reporting Items of Systematic Reviews and Meta-Analyses (PRISMA) flow diagram demonstrating the process of study selection in the meta-analysis. NAFLD: non-alcoholic fatty liver disease, TMAO: trimethylamine N-oxide.

We identified 136 studies during the initial database search (Pubmed: 60, Scopus: 73, Cochrane library: 3) (Figure 1). Following the removal of duplicates, we completed an Abstract and title screening of the remaining 79 studies. Fifty-nine studies were deemed ineligible due to their type (review/editorial) or the presence of preclinical information. The full text evaluation of the 20 records led to the subsequent exclusion of 13 studies due to the preclinical study design, the lack of a NAFLD group or TMAO data, or the overlapping study population. Finally, seven studies were considered eligible for data extraction. It should be noted that the study of Chen et al. consisted of two separate sub-studies, including a case-control and a cross-sectional study [20].

### 3.2. Study Characteristics

The characteristics of the included studies are presented in Table 1. From the seven included studies, we identified 2923 individuals with NAFLD and 4660 control subjects. Four studies had a cross-sectional design and two were case-control studies. One study consisted of a case-control and a cross-sectional part. Most studies were performed on Asian populations (4/7), whereas three studies concerned European subjects. NAFLD diagnosis was set via diagnostic scores (fatty liver index or hepatic steatosis index) in three studies or via liver ultrasound in two studies. Two studies used liver histology to document NAFLD. Regarding the method of TMAO measurement, most studies used high-performance liquid chromatography. One study used nuclear magnetic resonance spectroscopy, whereas another used early gas chromatography–mass spectrometry. Information regarding study characteristics was not available for the study of Moradzad et al. [21].

Only one study reported the odds ratio (OR) of TMAO levels with the presence of NAFLD [20]. This study included a case-control sub-study and a cross-sectional sub-study. In its case-control sub-study, the Ln(TMAO) levels were found to predict the presence of any degree of steatosis (OR 3.58, 95% CI 1.38 to 2.96, *p* < 0.009). In its cross-sectional sub-study, the highest TMAO quartile had a trend toward an association with any degree of NAFLD (OR 1.53, 95% CI 1.08 to 2.17, *p* = 0.11). However, this association was more potent when moderate/severe NAFLD was set as the variable of interest (OR 2.62, 95% CI 1.53 to 4.50, *p* = 0.004).

### 3.3. Meta-Analysis

**Figure 2:**
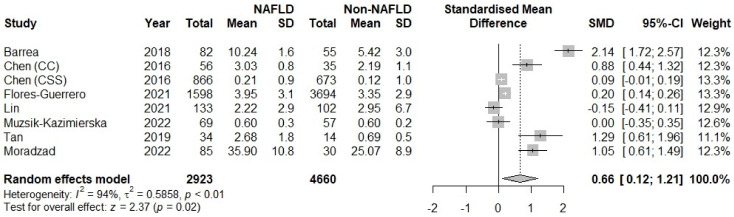
Forest plot displaying the meta-analysis of circulating trimethylamine N-oxide (TMAO) difference between individuals with and without non-alcoholic fatty liver disease (NAFLD), demonstrating significantly higher TMAO levels in the NAFLD group. Effect sizes were pooled according to the random-effects model. I^2^ was used as a measure of between-study statistical heterogeneity. Results are expressed as standardized mean difference (SMD) with horizontal error bars denoting the 95% confidence intervals (CIs). The size of each square represents the relative weight of that study in the overall meta-analytic result.

**Figure 3:**
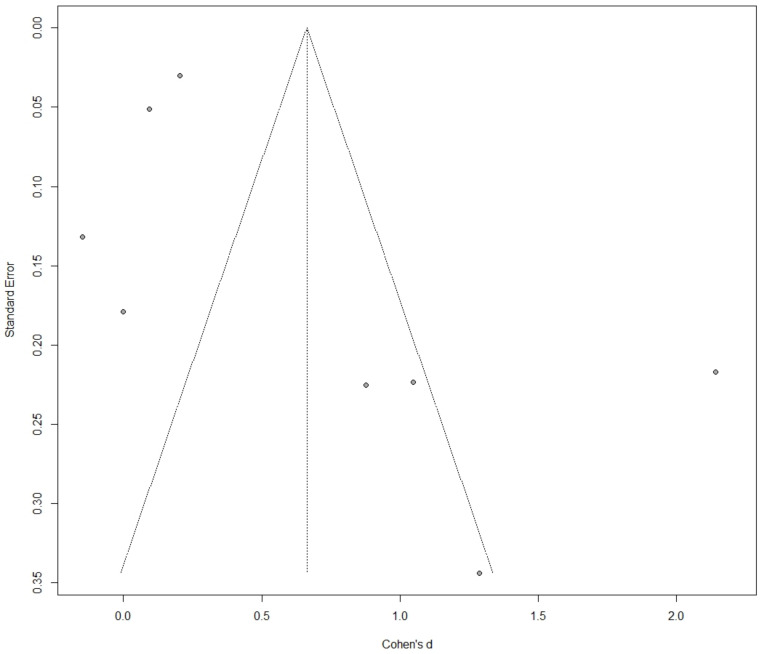
Inspection of an asymmetric funnel plot of the difference in trimethylamine N-oxide (TMAO) between the non-alcoholic fatty liver disease (NAFLD) and control subjects. Cohen’s d was used as the effect size metric plotted against the standard error.

Based on the results of our meta-analysis, individuals with NAFLD had significantly higher levels of circulating TMAO compared to the control groups (SMD: 0.66, 95% CI −0.12 to 1.21, *p* = 0.02) (Figure 2). Substantial between-study heterogeneity was noted (I^2^ = 94%). One study was identified as influential. Its exclusion led to a small reduction of the between-study heterogeneity (I^2^ = 87%) and the overall effect (SMD: 0.43, 95% CI 0.02 to 0.85, *p* = 0.04). The funnel plot inspection revealed asymmetry, indicative of publication bias (Figure 3). The overall risk of bias was considered moderate (Table 2).

**Figure 4:**
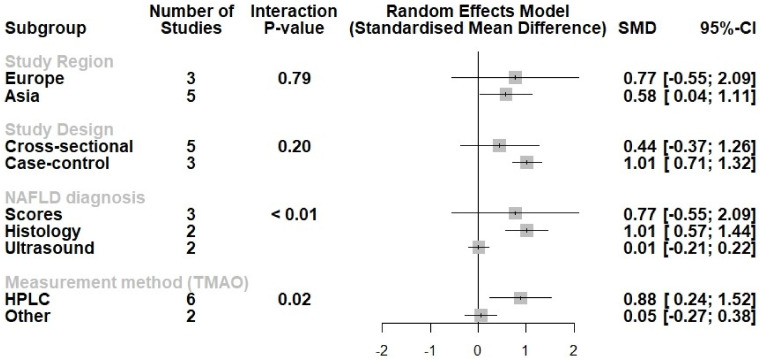
Subgroup analysis displaying no differences according to the study design or study region. Effect sizes were pooled according to the random effects model and the subgroup analysis followed the fixed-effects (plural) model. Results are expressed as standardized mean difference (SMD) with horizontal error bars denoting the 95% confidence intervals (CIs).

Finally, we conducted a subgroup analysis (Figure 4). We noted no differences in the association between NAFLD and circulating TMAO according to the study region (Europe/Asia) or the study design (cross-sectional/case-control). Most importantly, we noted a more potent association between NAFLD and circulating TMAO in studies that utilized liver biopsy as the method of NAFLD diagnosis (*p* for interaction <0.001), as well as in studies that measured TMAO with HPLC (*p* for interaction = 0.02).

## 4. Discussion

In this systematic review and meta-analysis of seven studies with 7583 individuals, we sought to address the interaction between gut dysbiosis and NAFLD by evaluating the association of a major gut dysbiosis metabolite, TMAO, with NAFLD. According to our results, individuals with NAFLD had significantly higher levels of TMAO compared to the controls. Despite the significant between-study heterogeneity that was observed, the results remained unaffected after removing the influential study. Additionally, we noted greater TMAO disparities between NAFLD and non-NAFLD patients in studies utilizing liver histology as the method of NAFLD diagnosis and HPLC as the method of TMAO measurement.

The gut microbiota is made up of a wide range of microorganisms that perform metabolic, synthetic, and regulatory tasks, such as the metabolism of bile acids, the fermentation of indigestible dietary substrates, the synthesis of vitamins, the control of epithelial cell proliferation, or the modulation of the inflammatory response [27]. By competing with pathogens for resources and available space, the gut microbiota also prevents pathogen colonization, acting as the intestinal barrier’s first line of defense. Genetic and environmental variables, including food, alcohol use, and the use of certain medicines, have a significant impact on the composition of the microbiota. The metabolic activity of the bacterial community is highly impacted by these variables, which also play a role in the etiology of a number of illnesses [28]. Dysbiosis and changes to the intestinal barrier are closely linked to inflammation and metabolic diseases [29]. Preclinical research has demonstrated that the microbiome of obese mice is more capable of obtaining energy from food and also affects how this energy is utilized and stored [30]. Low bacterial diversity and distinctive metagenome changes in humans enable the identification of subgroups of people with high-risk metabolic profiles as well as microbiome signatures for associated disorders including type 2 diabetes mellitus and obesity [31,32].

The increasing trends in NAFLD incidence signify the importance of its prompt recognition to avoid possible hepatic and extrahepatic complications. Moreover, the pathophysiologic basis of NAFLD is multifactorial and incompletely understood. The most potent contributor to NAFLD development appears to be insulin resistance [33]. This process is regulated by reduced levels of high molecular weight adiponectin and the vagus nerve. Adiponectin exerts its protective effect via the regulation of AMP-activated protein kinase activity and an increase in the expression of peroxisome proliferator-activated receptor (PPAR)-α target genes (CD36, acylcoenzyme A oxidase, and uncoupling protein 2). The vagus nerve protects against NAFLD via modulation of peroxisome proliferator-activated receptors (PPAR) and peripheral lipolysis. In insulin resistance states, an abundance of free fatty acids in the liver is observed, possibly due to de novo lipogenesis (DNL) (via the sterol regulatory element binding protein 1c (SREBP1c) and the carbohydrate response element binding protein (ChREBP)) and lipoprotein uptake. Chronic hyperinsulinemia also reduces apolipoprotein B100 production and, as a result, VLDL-associated lipid export from liver cells. Thus, hyperinsulinemia promotes hepatic lipid synthesis while inhibiting triglyceride release as VLDL (steatosis) [34]. Furthermore, FFAs in the liver promote lipid peroxidation, the generation of highly reactive oxygen species, and the release of proinflammatory cytokines such as the tumor necrosis factor-α, which promotes inflammatory processes and liver fibrosis (steatohepatitis). Toxic metabolites such as diacylglycerol and ceramides can cause insulin resistance during DNL, resulting in a positive feedback loop in which insulin resistance encourages hepatic DNL and hepatic DNL promotes insulin resistance [35].

Recently, the interaction of the gut microbiome with various pathologic states has been introduced. In particular, the gut-liver axis and TMAO appear to be essential in NAFLD pathogenesis, in addition to the established pathophysiologic concepts. TMAO may be related to the common NAFLD risk factors such as type 2 diabetes mellitus, obesity, and hypertension, among others, as depicted by recent meta-analyses [36,37,38,39]. Additionally, an early experimental study pointed to the association of TMAO with insulin resistance as a driver of NAFLD [40]. TMAO may affect the development of NAFLD through other distinct mechanisms. Food that is high in trimethylamine precursors, such as red meat, eggs, and fish, are metabolized by the digestive system into choline, L-carnitine, betaine, and γ-butyrobetaine. Gut bacteria convert the excessive TMA precursors that cannot be absorbed into TMA. Flavin monooxygenases (FMO) 1 and 3, produced by the liver, then convert trimethylamine into TMAO after it has been absorbed into the circulation via the intestinal mucosa. A recent study by Shi et al. highlighted the hazardous interaction of TMA with FMO [41]. In vitro, high fat exposure by stimulation of L02 cells with oleic acid and palmitate enhanced FMO1 mRNA expression and TMAO concentration, as well as free fatty acid and triglyceride content. Along the same lines, they also found a dose-dependent increase in the above parameters with TMA stimulation of L02 cells. Moreover, by influencing oxidative stress, TMAO can directly enhance the onset of NAFLD [42]. Another important concept consists of unfolded protein response activation by gut dysbiosis and TMAO. This could result in disordered hepatocyte lipid metabolism, inflammation, and ultimately death, aiding NAFLD development and progression [43,44]. Preclinically, TMAO stimulation resulted in an increased expression of unfolded protein response-related proteins (GRP78, XBP1, and Derlin-1) [41], further supporting this hypothesis. Finally, TMAO may enhance the risk of fatty liver disease by lowering the total bile acid pool size through the reduction of bile acid production via the following: (1) the suppression of the important enzymes CYP7A1 and CYP27A116 and (2) the restriction of bile acid enterohepatic circulation by repression of the organic anion transporter and the expression of the multidrug resistance protein family [45]. Bile acids, in addition to their well-known involvement in dietary lipid absorption and cholesterol balance, operate as metabolically active signaling molecules that modulate glucose and lipid metabolism. As a result, it is likely that TMAO may alter hepatic TG levels and cholesterol transport, glucose and energy balance, and bile acid production and transport, indicating that TMAO is a potential risk factor for NAFLD.

The deleterious effect of TMAO was identified in an experimental mouse model treated with 3% high choline water [46]. This animal model exhibited a significant weight gain and impaired endothelial function evidenced by increased endothelin-1 and a decreased nitric oxide concentration. Moreover, high choline water led to abnormalities in the lipid profile (i.e., an increase in serum low-density lipoprotein cholesterol, total cholesterol, and triglycerides and a decrease in serum high-density lipoprotein) and liver function tests (i.e., an increase in aspartate and alanine transaminases). An interesting observation was the increased liver oxidant enzymes (malondialdehyde and non-esterified fatty acid) and the decreased antioxidant defenses (superoxide dismutase and glutathione peroxidase). Finally, high choline water induced significant histological changes with hematoxylin and eosin staining (severe cellular degeneration, massive fatty changes, hepatocyte necrosis, and loss of cellular boundaries), indicative of fatty liver disease. This finding was accompanied by evidence of a widespread deposition of lipid droplets inside the parenchyma cells with Oil Red O staining. Overall, this experimental study suggests that a high choline regimen could promote liver injury in the form of NAFLD, with TMAO upregulation being a speculated mechanism. The proposed influential role of gut dysbiosis and TMAO in NAFLD pathogenesis could suggest that treatments that directly inhibit TMAO synthesis (3,3-dimethyl-1-butanol, iodomethylcholine, or fluoromethylcholine) may be effective in ameliorating NAFLD. However, this should be tested further in future preclinical studies.

Our systematic review and meta-analysis are mainly limited by the substantial between-study heterogeneity. This could be attributed to numerous factors, such as the various NAFLD definitions, the characteristics of the control group, and the method of TMAO measurement, among others. Moreover, the existence of publication bias and the overall medium methodological quality of the included studies must also be considered. However, our results represent an important piece of evidence supporting the deleterious interaction of gut dysbiosis with the liver. Future clinical studies should further assess the impact of gut microbial metabolites in NAFLD and the therapeutic potential of their inhibition.

## 5. Conclusions

In this systematic review and meta-analysis, patients with non-alcoholic fatty liver disease had increased levels of trimethylamine N-oxide, a hazardous gut microbial metabolite, suggesting its important role in the gut–liver interaction.

## Figures and Tables

**Table 1:** Characteristics of the studies included in the meta-analysis.

Study	Study Design	Year	Region	NAFLD Definition	TMAO Measurement	N (NAFLD)	N (Control)
Barrea [22]	Cross-sectional	2018	Europe	FLI >30	HPLC	82	55
Chen [20]	Case-control	2016	Asia	Histology	HPLC HPLC	56	35
	Cross-sectional			Ultrasound		866	673
Flores-Guerrero [23]	Cross-sectional	2021	Europe	FLI ≥30	NMR spectroscopy	1598	3694
Lin [24]	Cross-sectional	2021	Asia	Ultrasound	Early gas chromatography–mass spectrometry	133	102
Muzsik-Kazimierska [25]	Cross-sectional	2022	Europe	HIS >36	HPLC	69	57
Tan [26]	Case-control	2019	Asia	Histology	HPLC	34	14
Moradzad [21]	Case-control	2022	Asia	NA	HPLC	85	30

NAFLD: non-alcoholic fatty liver disease, TMAO: trimethylamine N-oxide, FLI: fatty liver index, HIS: hepatic steatosis index, HPLC: high-performance liquid chromatography, NMR: nuclear magnetic resonance, NA: not available.

**Table 2:** Risk of bias assessment according to the Newcastle–Ottawa Quality assessment scale.

Study	Newcastle–Ottawa Quality Assessment Scale				
	Selection	Comparability	Exposure/Outcome	Score	Quality
Barrea [22]	***	-	***	6/10	Moderate
Chen (CC) [20]	***	-	**	5/9	Moderate
Chen (CS) [20]	***	-	***	6/10	Moderate
Flores-Guerrero [23]	*****	-	***	8/10	High
Lin [24]	***	-	***	6/10	Moderate
Muzsik-Kazimierska [25]	**	-	***	5/10	Moderate
Tan [26]	***	**	**	7/9	High
Moradzad [21]	NA	NA	NA	NA	NA

CC: case-control, CS: cross-sectional, NA: not available. Number of * represents scoring points in each category.

## Supplementary Materials

The following supporting information can be downloaded at: https://www.mdpi.com/article/10.3390/metabo12121243/s1, Table S1: Preferred Reporting Items of Systematic Reviews and Meta-Analyses (PRISMA) statement checklist.
